# Anti-monopoly supervision model of platform economy based on big data and sentiment

**DOI:** 10.3389/fpsyg.2022.953271

**Published:** 2022-07-28

**Authors:** Sihan Liu

**Affiliations:** School of Economics, Liaoning University, Shenyang, China

**Keywords:** big data, sentiment analysis, platform economy antitrust supervision model, vector space model, model of economic anti-monopoly supervision

## Abstract

With the advent of the cloud computing era, big data technology has also developed rapidly. Due to the huge volume, variety, fast processing speed and low value density of big data, traditional data storage, extraction, transformation and analysis technologies are not suitable, so new solutions for big data application technologies are needed. However, with the development of economic theory and the practice of market economy, some links in the industrial chain of natural monopoly industries already have a certain degree of competitiveness. In this context, the article conducts a research on the anti-monopoly supervision mode of platform economy based on big data and sentiment analysis. This paper introduces the main idea of MapReduce, the current software implementation specifies a Map function that maps a set of key-value pairs into a new set of key-value pairs. It specifies a concurrent Reduce function that guarantees that each of all mapped key-value pairs share the same set of keys. establishes a vector space model, and basically realizes the extraction of text emotional elements. It introduces the theoretical controversy of antitrust regulation of predatory pricing behavior of third-party payment platforms, and conducted model experiments. The experimental results show that the throughput of 40 test users in 1 h of test is determined by two factors, QPS and the number of concurrent, where QPS = 40/(60*60) transactions/second. The time for each test user to log in to the system is 10 min, and the average response time is 10*60 s, then the number of concurrency = QPS*average response time = 40/(60*60)*10*60 = 6.66. This paper has successfully completed the research on the anti-monopoly supervision model of platform economy based on big data and sentiment analysis.

## Introduction

With the advent of the era of big data, the world is facing the challenge of unprecedented growth of information, and Internet information processing technology has also ushered in rapid development. The method of interconnecting computer networks may be referred to as “interworking.” On this basis, a global Internet network covering the whole world is developed, called the Internet, which is a network structure that is connected to each other. How to accurately, timely and effectively obtain the emotional information of short texts on the Internet is the driving force for the harmonious, stable and orderly development of the society, and has important value and significance. However, Internet short texts are free, flexible and lack normative, and the emotional information in them is affected by time, place, environment, and emotional scenes triggered by characters, all of which make it difficult for traditional text sequence labeling methods to identify emotional elements; secondly, most of the existing text emotional analysis only stays at the surface analysis such as classification, but does not identify the root cause of text emotional events.

Antitrust is the prohibition of monopolies and trade restrictions. It is an intervention method adopted by national governments or international organizations when a company’s marketing presents a monopoly or a tendency to monopolize. According to traditional anti-monopoly law theory, we should determine predatory pricing behavior from three aspects: whether there is a market structure that is favorable to predators; whether there is a pricing behavior below cost; if we can make up for the loss during the low price period, and get the monopoly profit after the successful robbery. Theoretically, the study of the predatory pricing behavior of agents is based on the two-sided market. In terms of bilateral market research, foreign countries focus on the external effects of network and pricing behavior, and the research results are relatively mature. It is believed that users on both sides of the two-sided market realize their usefulness on the basis of the increasing size and number of the other side, and there is an external effect between the two. In order to internalize the external effects of the two-side market network, an asymmetric price structure is adopted, and a low-cost or free consumer pricing model is adopted, thereby increasing the enthusiasm of traders to enter the platform business.

## Related work

Text sentiment data is another embodiment of big data, and the Internet platform can serve as a new carrier for people to express their opinions, ideas and opinions. Salimi M determined the effectiveness of group-based acceptance and commitment therapy for cognitive emotion regulation strategies in mothers of children with autism. He selected 30 mothers of children with autism spectrum disorders and randomly divided them into two groups: an experimental group and a control group. However, the sentiment analysis results and frame structure he studied are not applicable to a wide range of occasions and have limitations (Salimi et al., 2019). Moreira M believes that affective-aware computing represents a development in machine learning that enables systems and devices to interpret emotional data to identify changes in human behavior. He proposed an improved algorithm for an emotion-aware intelligent system capable of predicting the risk of postpartum depression in women with hypertensive disorders of pregnancy through biomedical and sociodemographic data analysis. It turns out that ensemble classifiers are the main solution for predicting pregnancy-related psychological disorders (Moreira et al., 2019). Based on big data and the theory of public opinion generation and dissemination, Li J built a big data-based employee public opinion system and its main functions, including big data collection, data processing, data storage, data analysis, data visualization and employee public opinion early warning. Finally, he defines the rich connotation of employee public opinion index (Li and Xu, 2020). Sun A built a software-defined network (SDN) multi-emotion cloud platform to connect different emotion clouds. By splicing the advantages of the control plane and data plane, the routing path can be changed using software, which means that individual cases of different students can be handled by a dedicated system through the Service Function (SF). However, his research did not promote the application of the built emotional cloud platform, and still has considerable limitations (Sun and Huang, 2019).

Industry regulation is carried out by independent regulators in order to safeguard the public interest of society. Using the methods of the French regulatory school, Montalban M described the nature and changes in the forms of competition inherent in platforms. He argues that this will accelerate some of the trends and institutional forms that characterize the financialization of RA, and that it is an endogenous product of its crisis. But his research did not take into account the dysfunction and possible conflicts due to the regulatory model (Montalban et al., 2019). Xg believed that the analysis of the regulatory environment is the first step for companies to enter the new electricity market. Transmission and distribution assets are the main investment targets. In this way, he conducted research on the economic supervision model of the electricity market and its incentive mechanism. His research showed that the current economic supervision model of the electricity market has certain limitations and is almost monopolized by several large enterprises, which is not conducive to the healthy competition of the market. But his research did not propose a practical solution (Xg et al., 2020). With the theme of “Dazhou Disaster Medical Rescue Skills Training Based on Virtual Simulation Platform,” He X analyzed the disaster medical rescue personnel training system based on virtual simulation platform from the aspects of training course structure, course arrangement, and assessment standards. And evaluated the training effect, he found out the problems in the training process. However, he did not propose any countermeasures to further improve the training in the future (He, 2022). Marco discussed the effectiveness of the regulatory framework established to safeguard competition in Hong Kong’s telecommunications industry. The results of the liberalization process are certainly notable, and the city’s telecommunications market is very competitive. However, it has been argued that enthusiasm for the outcome of the liberalization process may have overshadowed important competition issues in local markets that could have been addressed through the development of strong antitrust policies (Marco Colino, 2019).

## Big data and market antitrust methods

### Main idea of MapReduce

**FIGURE 1:**
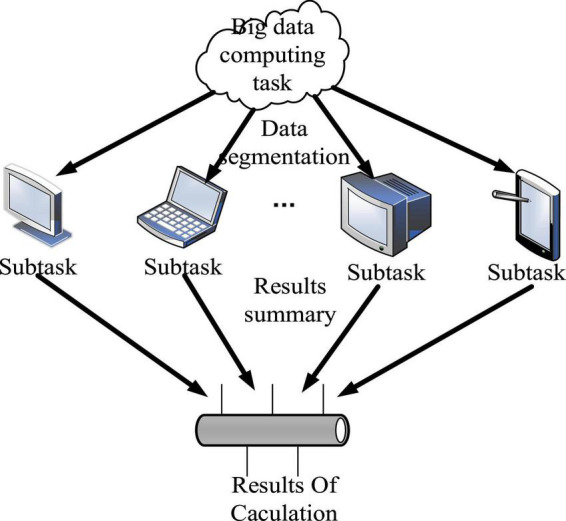
The main idea of the parallel programming model.

The MapReduce parallel programming model proposed by Google and its open source implementation in Apache Hadoop has become the main model for big data processing because of its simplicity, scalability and fault tolerance (Barnett, 2019). The core idea of the parallel programming model is to divide and conquer, that is, by slicing dense big data with no inherent dependencies into multiple pieces. It is calculated and processed in parallel by multiple subtasks, and finally the results are aggregated to the control task for output (Agnese, 2018). The whole process is shown in Figure 1.

**FIGURE 2:**
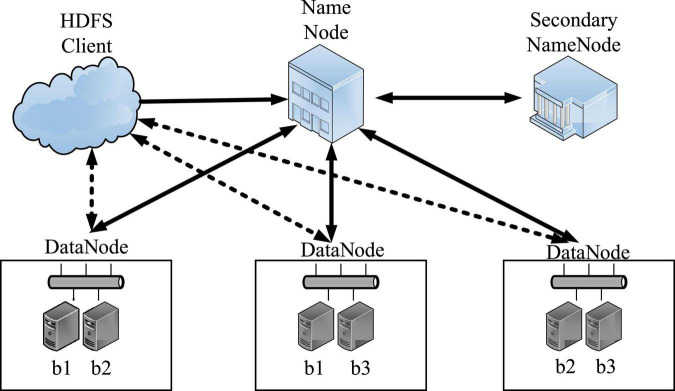
Basic architecture of HDFS.

Hadoop Distributed File System (HDFS) is a distributed file system under the Hadoop open source project, which is designed based on the streaming data access mode, and is widely used for big data file storage management (Katz, 2019). It is a highly fault-tolerant system suitable for deployment on inexpensive machines. HDFS can provide high-throughput data access, which is very suitable for applications on large-scale data sets. In the design of the basic architecture, HDFS adopts the master-slave node and backup node mode (Derave et al., 2022), which ensures the high reliability, high scalability, and high fault tolerance of the storage cluster (Munaiseche et al., 2022). Its basic architecture is shown in Figure 2.

The large data files of HDFS are divided into multiple data blocks and stored in multiple data nodes. The default size of the data block is 128MB (Wu et al., 2018). Each data block will have 3 copies by default for data backup, which are the local node data block and two cross-rack and different node data blocks (Huda et al., 2017). Such a multi-copy strategy ensures that HDFS can obtain the best read and write performance and optimize data localization while reducing the risk of data invalidation or loss caused by data node failure (Zhang and Jin, 2021). HDFS adopts a master-slave (Master/Slave) structure model. An HDFS cluster consists of a NameNode and several DataNodes. The NameNode acts as the main server to manage the namespace of the file system and the client’s access to files; the DataNode in the cluster manages the stored data.

### Vector space model

The vector space model can be successfully applied to the well-known SMART text retrieval system. The processing of text content is simplified to vector operations in vector space, and it expresses semantic similarity by spatial similarity, which is intuitive and easy to understand. When doing dependency syntax, it is necessary to preprocess the existing training set, such as word segmentation, and learn a dependency syntax analysis function through training (Xu et al., 2022). When the dependency syntax analysis is required, the vector information obtained by preprocessing is compared with the obtained model (Xu, 2022), and the calculated data results of the two are used as the theoretical basis for judging the relationship between the two (Fu and Li, 2022). The above introduction shows that the word vector corresponds to the point in the space, and then is converted into a calculation in space. For the similarity, the distance between two points in the available space is generally expressed as follows (Xue et al., 2020):

Inner product formula:


(1) $$\mathrm{sim}\,\left({\text{d}}_{\text{i}},{\text{d}}_{\text{j}}\right)=\sum_{\text{k}=1}^{\text{n}}{\text{w}}_{\text{ik}}\times {\text{w}}_{\text{jk}}$$

The formula for the absolute value of the distance between two points:


(2) $$\mathrm{sim}\,\left({\text{d}}_{\text{i}},{\text{d}}_{\text{j}}\right)=\sum_{\text{k}=1}^{\text{n}}\left|w_{\text{ik}}-{\text{w}}_{\text{jk}}\right|$$

Euclidean distance, which is called the Pythagorean metric in earlier literature:


(3) $$\mathrm{sim}\,\left({\text{d}}_{\text{i}},{\text{d}}_{\text{j}}\right)=\sum_{\text{k}\,1}^{\text{n}}\left(w_{\text{ik}}-{\text{w}}_{\text{jk}}\right)$$

Chebyshev distance:


(4) $$\mathrm{sim}\,\left({\text{d}}_{\text{i}},{\text{d}}_{\text{j}}\right)=\text{max}\,\left|w_{\text{ik}}-{\text{w}}_{\text{jk}}\right|$$

Cosine of included angle:


(5) $$\mathrm{sim}\,\left({\text{d}}_{\text{i}},{\text{d}}_{\text{j}}\right)=\cos\theta =\begin{array}{c} \sum_{\text{k}=1}^{\text{n}}{\text{w}}_{\text{ik}}\times {\text{w}}_{\text{jk}}\\ \sum_{\text{k}=1}^{\text{n}}{\text{w}}_{\text{ik}}^{2}\,\sum_{\text{k}=1}^{\text{n}}{\text{w}}_{\text{jk}}^{2} \end{array}$$

Probability calculation includes addition rule, conditional probability, multiplication formula and total probability formula. Assuming that a given sequence is known to label the random variable X, the conditional probability distribution P Y(| X) of the random variable Y of the labeling result sequence is known, and the conditional probability P Y(| X) is the maximum. The existing probability formula is as follows:


(6) p(yi|x,∂)=1Z⁢(x)exp(∑j∂tjj(yi-1,yi,x,i)+∑kμskk(yi,x,i))

(7) Z⁢(x)=∑yexp⁢(∑j∂⁡tjj⁢(yi-1,yi,x,i)+∑kμ⁢skk⁢(yi,x,i))

**FIGURE 3:**
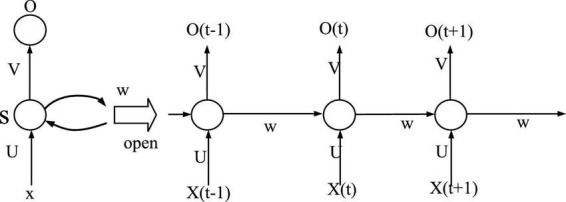
RNN expansion diagram.

Recurrent Neural Network (RNN) is a neural network model designed for sequence data, which has shown strong advantages in both text modeling and machine learning. Recurrent neural network is a type of recurrent neural network that takes sequence data as input, performs recursion in the evolution direction of the sequence, and connects all nodes (recurrent units) in a chain. The vocabulary that constitutes the RNN data, each word is randomly initialized. As the parameters of the model, a nonlinear prediction model is used to assign weights to it, as shown in Figure 3.

Among them, the input layer input sequence is marked as:


(8) $$\left\{{\text{x}}_{0},{\text{x}}_{1},\ldots \,\ldots ,{\text{x}}_{\text{t}},{\text{x}}_{\text{t}+1},\ldots \,\ldots \right\}$$

The output information sequence of the hidden layer is marked as:


(9) $$\left\{{\text{s}}_{0},{\text{s}}_{1},\ldots \,\ldots ,{\text{s}}_{\text{t}},{\text{s}}_{\text{t}+1},\ldots \,\ldots \right\}$$

The output information sequence of the output layer is marked as:


(10) $$\left\{{\text{y}}_{0},{\text{y}}_{1},\ldots \,\ldots ,{\text{y}}_{\text{t}},{\text{y}}_{\text{t}+1},\ldots \,\ldots \right\}$$

LSTM (Long Short-Term Memory) is an extended network model in which RNN redesigns the memory module of the hidden layer nodes to build a special memory storage unit. It adds an Input Gate and an Output Gate to adjust the information of the input data and the state information of the memory unit, which is suitable for context-related text analysis tasks. The following is the calculation formula for the structure of the LSTM model:


(11) $$i_{t}=\sigma \,\left(W_{i}\,x_{i}+U_{i}\,h_{i-1}+b_{i}\right)$$

(12) $$f_{t}=\sigma \,\left(W_{f}\,x_{t}+U_{f}\,h_{i-1}+b_{f}\right)$$

(13) $$o_{t}=\sigma \,\left(W_{o}\,x_{i}+U_{o}\,h_{i-1}+b_{o}\right)$$

(14) $$g_{t}=t\,a\,n\,h\,\left(W_{c}\,x_{i}+U_{c}\,h_{i-1}+b_{c}\right)$$

(15) $$c_{t}=f_{i}\,ℶ\,C_{I-1}+I_{1}\,ℶ\,G_{t}$$

In summary, the extraction of text emotional elements is basically realized.

### Theoretical controversy over anti-monopoly laws on predatory pricing by third-party payment platforms

The third-party payment industry is a two-sided market that is prone to oligopoly, and influential third-party payment platforms often occupy a high market share, such as Alipay. In such an environment, the impact of market share on market power will be weakened. In contrast, the traditional market structure criteria for determining market power should be improved, and it is no longer possible to judge whether an enterprise has a dominant position based on market share alone, and the proportion of market share should be appropriately increased. Its characteristics are: 1. It is basically a homogeneous product, such as basic chemicals or gasoline. 2. Relatively few sellers, such as some large companies and many small companies that follow large companies. 3. Demand curves in apparently inelastic industries. When calculating market share, traditional calculation methods cannot accurately reflect the real situation of the market. Because there are a large number of products provided to users for free in a two-sided market such as a third-party payment platform, the basis and meaning of the calculation will be lost when the formula is used for calculation. Moreover, the traditional formula for calculating market share is based on sales, but in the field of third-party payment, due to the existence of positive feedback effects, the market share of platform companies is more expressed by the number of sales. The number of sales can more directly reflect the ability of platform companies to control the market. Therefore, in the third-party payment platform industry, sales cannot be directly used as an indicator for determining market share, but data such as user usage scale should be included in the scope of reference. Specifically, when calculating market share, it can be determined by dividing the number of users of the platform by the total number of users of alternative platforms in the market.

## Experiments on the anti-monopoly supervision model of the platform economy

### Economic efficiency of time division multiplexing synchronous code division multiple access patent alliance

The Chinese meaning of TD-SCDMA is Time Division Multiplexing Synchronous Code Division Multiple Access. It was first proposed by China, completed on the basis of Radio Transmission Technology (RTT), and has officially become an international mobile communication standard accepted by ITU. This is a pioneering work in China’s mobile communication industry and a contribution to the international mobile communication industry, as well as an unprecedented breakthrough made by China in the field of mobile communication.

A patent alliance is an alliance between enterprises based on common strategic interests and linked by a group of related patented technologies. Enterprises within the alliance realize cross-licensing of patents, or mutually preferentially use each other’s patented technologies, and jointly issue a joint license statement to the outside of the alliance.

**FIGURE 4:**
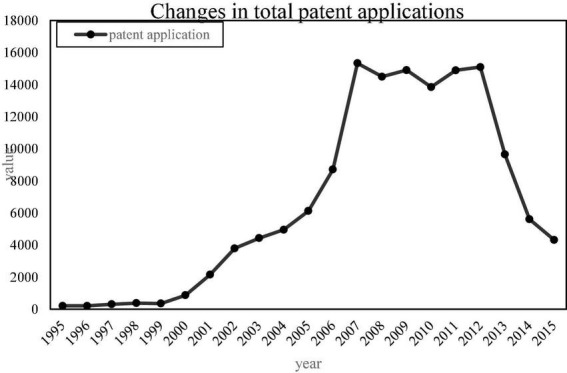
Changes in the total number of patent applications of several major members before and after the establishment of the Time Division Multiplexing Synchronous Code Division Multiple Access Patent Alliance.

Since there are many types of patents, and the scope of patents involved in enterprises is wide, we only need to count the patents related to the TD-SCDMA standard. According to the IPC international classification number, most of the mobile communication patents are distributed in the H04L, H04Q, H04W, H04B7, H04J, and H04M categories. Therefore, this article mainly searches for H04 patents, and sorts out the patent application data of several main members of the alliance, Huawei, ZTE, Lenovo, China Mobile and Samsung Electronics. However, the patent application data in 2017 was not updated completely, and the official website of the State Intellectual Property Office was used to sort out the total number of patent applications of several major members in the alliance between 1995 and 2016, as shown in Figure 4.

As can be seen from Figure 4, after the establishment of the TD-SCDMA Patent Alliance, the number of H04 patents of members has grown rapidly. Although there is a downward trend in the middle, the number of patent applications each year is still at a high level. After the establishment of the patent alliance in 2002, the number of H04 patent applications increased by 55%, which shows that the patent alliance can promote the innovative behavior of the members in the alliance. In order to verify this, this paper conducts the Wilcoxon rank sum test on the number of patent applications before and after the establishment of the patent alliance. The null hypothesis is that the innovation behaviors before and after the formation of the alliance come from the same population and have the same distribution. If the result rejects the null hypothesis, then it is considered that the innovation behavior before and after the formation of the alliance comes from different populations, indicating that the formation of a patent alliance has an impact on the innovation behavior of enterprises.

**FIGURE 5:**
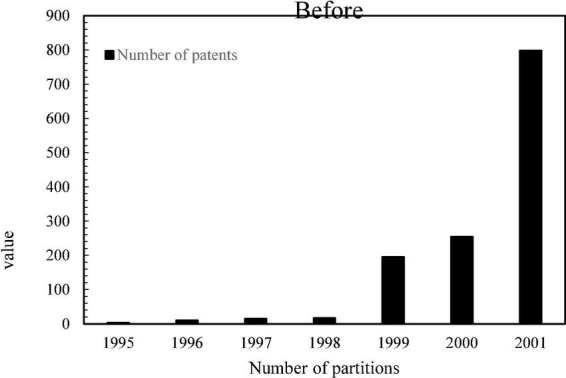
The number of H04 patent applications before a company joined the patent alliance.

Figure 5 shows the histogram of the number of H04 patent applications before an enterprise joined the patent alliance.

**FIGURE 6:**
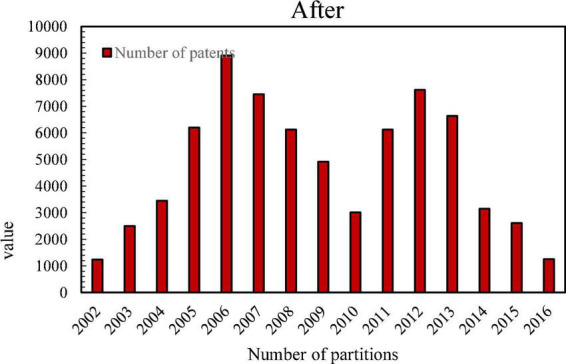
The number of H04 patent applications before and after a company joined the patent alliance.

Figure 6 shows the histogram of the number of H04 patent applications after an enterprise joined the patent alliance.

It can be seen that after a certain enterprise joins the TD-SCDMA Patent Alliance, its H-type patent application has been affected, and it is positive. Therefore, the establishment of a patent alliance will improve the innovation incentives of members in the alliance.

### Anti-monopoly and P2P online lending platform

**FIGURE 7:**
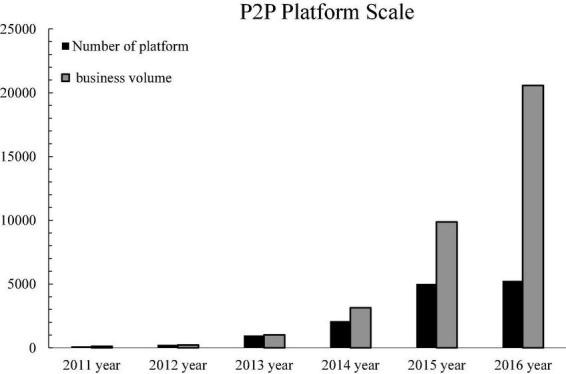
The scale of P2P platforms in China from 2011 to 2016.

The development of China’s P2P online lending platform has only gone through 10 years. The scale of P2P online lending platform has expanded rapidly and steadily increased. P2P online lending has been fully affirmed by the market with its unique advantages. The platform expansion area gradually extends from east to west, and the distribution of cities is tilted from first- and second-tier cities to third- and fourth-tier small cities. The total market financing has increased sharply from tens of millions to 100 billion yuan, as shown in Figure 7.

At present, the dust of the new regulatory policy has been settled, and the new policy strictly regulates P2P online lending platforms in terms of market access, business scale, and fund depository. They will face the bottleneck of shock adjustment, various problem platforms frequently occur, the growth rate of institutions will slow down, and the market development will gradually become more rational.

**FIGURE 8:**
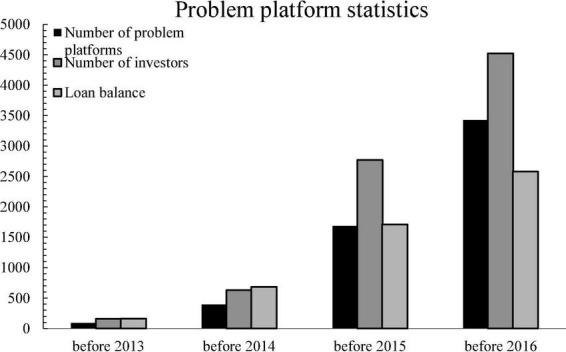
Problem platform statistics.

In recent years, the number of problematic platforms in China has been increasing year by year. The rapid growth of P2P online lending is accompanied by a sharp increase in risks, as shown in Figure 8.

As shown in the Figure 8, the cumulative number of problematic platforms in China is 275, accounting for 24% of the total number of registered platforms. As of the end of December 2015, the cumulative number of problematic platforms in China was 1,439, 2.47 times that of 2014, accounting for 33.2% of the total number of registered platforms. As of the end of December 2016, there were 3,429 problematic platforms in China, accounting for 58% of all registered platforms.

On the one hand, affected by the slowdown in macroeconomic growth, P2P online lending institutions frequently have overdue loans, extended loans, and more and more people are unable to repay their loans. In order to protect their own income, a large number of investors withdraw their own funds one after another, which breaks the funds of the P2P platform and is unable to cash it, which in turn triggers events such as business closure and running away. On the other hand, subjective reasons such as irregular operation of the platform itself and insufficient risk management and control capabilities make it difficult for the platform itself to maintain its operations.

### Emotional fuzzy pattern extraction of users and events

There are two ways for emotion recognition, one is to detect physiological signals such as respiration, heart rate and body temperature, and the other is to detect emotional behaviors such as facial feature expression recognition, speech emotion recognition and gesture recognition. The fuzzy pattern extraction of sentiment aims to mine the sentimental community sentiment in monopoly regulatory pattern events. Users with similar emotions in the community are likely to form emotional communities. The emotions of these communities do not only have a strong value in one dimension, but are significant in some dimensions. Mining the fuzzy patterns of emotions can find the main emotional communities in disordered emotions, and then conduct effective analysis. Emotion fuzzy pattern mining is mainly divided into three parts: establishing fuzzy similarity matrix, mining fuzzy pattern features and fuzzy decision. The fuzzy similarity matrix satisfies reflexivity and symmetry.

This paper selects some vocabulary, randomly invites 10 experts to determine the emotional tendency of the selected vocabulary, and obtains the initial matrix of vocabulary emotional tendency, as shown in Table 1.

**TABLE 1:** Lexical sentiment tendency initialization matrix.

W	S					
	
	Love	Joy	Surprise	Anger	Sadness	Fear
Acceptance	4	5	3	1	0	1
Anticipation	4	1	3	1	0	0
Courage	4	2	2	1	0	1
Dejection	1	0	1	2	4	2
Desire	4	3	2	0	0	1
Despair	0	0	1	1	5	2
Rage	0	0	1	5	2	1
Anxiety	1	1	1	1	3	2
Grief	1	0	0	0	5	0
Subjection	2	3	1	0	0	2
Expectancy	2	2	4	0	0	1
Panic	0	1	0	1	3	2

The fuzzy equivalence matrix of lexical sentiment tendency is shown in Table 2.

**TABLE 2:** Fuzzy equivalent matrix of lexical sentiment tendency.

w										
1	0.9622	0.9346	0.9711	0.9346	0.9128	0.9346	0.8986	0.9404	0.9428	0.9346
0.9622	1	0.9346	0.9622	0.9346	0.9128	0.9346	0.8986	0.9404	0.9428	0.9346
0.9711	0.9622	0.9346	0.991	0.9346	0.9128	0.9346	0.8986	0.9404	0.9428	0.9346
0.9346	0.9346	1	0.9346	0.9839	0.9128	0.9864	0.8986	0.9346	0.9346	0.986
0.9711	0.9622	0.9346	1	0.9346	0.9128	0.9346	0.8986	0.9404	0.9428	0.9346
0.9346	0.9346	0.9839	0.9346	1	0.9128	0.9839	0.8986	0.9346	0.9346	0.9839
0.9128	0.9128	0.9128	0.9128	0.9128	1	0.9128	0.8986	0.9128	0.9128	0.9128
0.9346	0.9346	0.9864	0.9346	0.9839	0.9128	1	0.8986	0.9346	0.9346	0.986
0.8986	0.8986	0.8986	0.8986	0.8986	0.8986	0.8986	1	0.8986	0.8986	0.8986
0.9404	0.9404	0.9346	0.9404	0.9346	0.9128	0.9346	0.8986	1	0.9404	0.9346
0.9428	0.9428	0.9346	0.9428	0.9346	0.9128	0.9346	0.8986	0.9404	1	0.9346
0.9346	0.9346	0.986	0.9346	0.9839	0.9128	0.986	0.8986	0.9346	0.9346	1

Then the fuzzy mode features are obtained as shown in Table 3.

**TABLE 3:** Fuzzy mode features.

P	S					
	
	Love	Joy	Surprise	Anger	Sadness	Fear
P1	0.9	0.52	0.7	0.12	0	0.4
P2	0.125	0.1	0.1875	0.25	0.75	1
P3	0	0	0.25	1	0.4	0.5
P4	0.25	0	0	0	1	0
P5	0.5	0.6	0.25	0	0	1

The fuzzy pattern mining algorithm divides the 12 words into five patterns: the first pattern is biased toward neutrality, the second type is biased toward negativity, the third type is overall low and biased toward negativeness, the fourth and fifth types of emotions are mixed to some extent, and the scales of the first and second categories are larger than those of the other categories. Assuming that the above pattern appears during the development of an event, it means that the development of the event has a good trend. On the whole, most of the vocabulary of the event is in a benign emotion. If the pattern of bad emotions has a high status in the event, then an early warning of the event should be issued.

In this part, this paper introduces in detail how to use fuzzy logic to analyze the sentiment of Weibo, so as to use the analysis results to control the development of events and provide effective suggestions. Sentiment analysis or opinion mining is the assessment of people’s opinions, sentiments, attitudes toward entities such as products, services, organizations, etc. The development and rapid start of this field benefit from the rapid development of social media on the Internet, such as product reviews, forum discussions, Weibo, WeChat, because this is the first time in human history that there is such a huge number of records.

To sum up, in the platform economic anti-monopoly supervision mode, the sentiment value and economic characteristics obtained by text sentiment analysis are introduced into regression algorithms for training. It proposes 2SA-MLR model, 2SA-STL model, 2SA-GM model, and 2SA-BPNN model. Then the above four single models are fused, and finally the 2SA-RERec model is proposed. Through the evaluation indicators MSE, RMSE, MAE and MAPE, the proposed 2SA-RERec model is compared with the other four single models, as shown in Table 4.

**TABLE 4:** Comparison of model experimental results.

	MSE	RMSE	MAE	MAPE
2SA-MLR	3430.960	58.574	23.990	0.0976
2SA-STL	3559.290	59.651	23.186	0.0825
2SA-GM	3481.427	59.004	22.016	0.0941
2SA-BPMN	**3428.732**	**58.555**	21.978	0.0828
**2SA-RERec**	3429.188	58.559	**21.608**	**0.0806**

The bold data represents the minimum value of the model, and the smaller the value, the higher the fitting accuracy of the model is.

Each row is the best result of the model proposed in this chapter in their respective experimental comparisons. It can be seen from the Table 4 that the structure of the neural network model is complex and the data training is sufficient. The results of the 2SA-BPNN model training are added to the initial prediction results to obtain the final predicted value, so the 2SA-RERec recommendation model performs better in terms of error. The MAE and MAPE values of the fused 2SA-RERec model are the smallest, indicating that the fitting accuracy of the model is better, and the prediction accuracy of the other four single emotion models is slightly worse. The MAPE values are all below 10%, indicating that multiple emotional features further improve the prediction accuracy and enhance the robustness of the model. Multiple sentiment features will further improve the prediction accuracy, indicating that the introduction of sentiment analysis technology is effective. Table 5 is the analysis of the transaction passing situation.

**TABLE 5:** Transaction pass analysis.

Affair	Adopt	Fail	Stop
Query	40	0	0
Newly added	40	0	0
Statistics	40	0	0
Subscribe	40	0	0
Delete	40	0	0
Page turning	40	0	0

**FIGURE 9:**
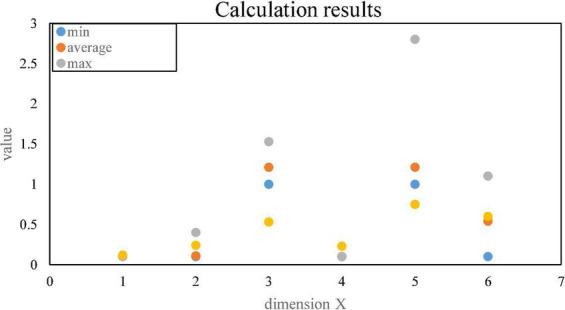
Transaction response time.

Figure 9 shows the transaction response time.

The throughput analysis is as follows: The throughput of 40 test users within 1 h of testing is determined by two factors, QPS and the number of concurrent transactions, where QPS = 40/(60*60) transactions/second. The time for each test user to log in to the system is 10 min, and the average response time is 10*60 s, then the number of concurrency = QPS*average response time = 40/(60*60)*10*60 = 6.66.

Analysis of server resource usage: CPU usage is 20% and memory usage is 10%, indicating that the sentiment analysis system performs well.

## Resolv of emotion algorithm

### Description of the effectiveness of the fusion algorithm

In the chapter of multi-physical domain information processing, many characteristic quantities describing multi-physical domain signals are introduced, and these characteristic quantities in time domain or frequency domain describe the change of the state of the measured object from different angles. Therefore, in the stage of feature extraction, it is desirable to obtain as many features as possible to use as model data. However, as the amount of features increases, the computational efficiency of the perception model will be reduced in both training and testing of the autonomous perception model, and there may be information overlap between different feature amounts. Therefore, it is necessary to reduce the extracted multi-physical domain features to reduce the redundancy of information. The next generation wireless network will be wireless personal area network (such as Bluetooth), wireless local area network (such as Wi-Fi), wireless metropolitan area network (such as WiMAX), public mobile communication network (such as 2G, 3G) and Ad Hoc network, etc.

According to human physiology, there are some primary areas on the two hemisphere cortex of the brain. After various sensory organs (eyes, ears, nose, etc.) are stimulated, signals are transmitted from the central nervous system to the corresponding areas. At this time, multiple feature detection cells with the same receptive field will gather together, and the comprehensive response of various stimuli in the same sensory pattern can be achieved. Such a simple perception is formed, and this fusion of the human brain is a multi-physical domain and multi-modal fusion of a two-layer structure. Multi-physical domain and multi-modal fusion is based on this mechanism, and the multi-modal features of multi-physical domain information are divided into multiple groups to form a fusion system with a two-layer structure. Multiple low-level features are fused by algorithms such as HMM that can perform time series modeling, and each result is fused by quadratic decision-making based on algorithms such as D-S and Bayesian. It first acquires and stores multi-physical domain data, and uses multi-physical information acquisition systems and multi-sensors to synchronously collect state data for sensing objects. Then, the data collected by each channel are cleaned and feature extracted, respectively. Afterward, the obtained features of each physical information are used for model training using the perceptual algorithm to obtain the perceptual model of each physical domain. Among them, various states corresponding to the model libraries of each physical domain need to be trained separately, and N physical domains correspond to N independent model libraries. Finally, the perception result of each physical domain information is used as the input of multi-physical domain information decision fusion, and the decision fusion is performed by using D-S evidence theory or decision fusion algorithms such as Bayesian.

### Vertical integration

For traditional industrial organization theory, its research on market structure is only limited to the analysis of behavioral efficiency among firms in horizontal markets, while the analysis of vertical markets is relatively rare. With the development of economy and society, in the process of judicial practice and anti-monopoly regulation, economists gradually realized the importance of vertical structure research, which mainly includes two types, namely vertical integration and vertical restriction.

Vertical integration refers to the specific combination of upstream and downstream manufacturers to form an integrated enterprise, including forward integration and backward integration. Forward integration means that upstream manufacturers take measures to merge with downstream manufacturers, while backward integration is the opposite.

In the market, manufacturers that sell final products need to obtain products from upstream manufacturers as inputs, and upstream manufacturers may also need to obtain products from their upstream manufacturers as inputs, thus forming a vertical supply chain. In such a chain, there are problems such as uncertainty, information asymmetry, and the respective monopoly power of upstream and downstream, and vertical integration came into being. Generally speaking, the motives for the formation of vertical integration are as follows: First, due to the transaction of final products from upstream to downstream, a series of links must be experienced in the middle. These links will cause certain efficiency losses, and vertical integration will internalize these efficiency losses. Second, the ability to obtain rents can be expanded; third, price discrimination can be implemented in the market.

### Counter-effects of restricting competition

The anti-monopoly laws of various countries recognize an important competition concern-blocking effect. The blocking effect in the sense of anti-monopoly law means that barriers to market entry are artificially set up to prevent new competitors from joining, so that the relevant market is closed. One of the legislative purposes of the Anti-Monopoly Law is to prevent the reckless behavior of operators with higher market power, use legal weapons to protect weak market players, and then ensure the smooth and orderly operation of the market competition mechanism. Studying the anti-monopoly history of various countries, the construction and improvement of their legal systems may be due to different social backgrounds, but they have the same social governance concept, that monopoly is harmful. Such arbitrary market behavior will seriously endanger the survival of vulnerable market individuals, and will also limit the free choice of consumers and the realization of welfare. The formulation of the Anti-Monopoly Law is to curb monopoly to achieve the goal of maintaining the market competition mechanism and protecting the legitimate interests of consumers. In commercial activities, buy-side companies with high market power usually want to use MFC terms to combat competitors, increase their transaction costs, and exclude existing or potential competitors outside the market. If new entrants are unable to attract buyers at competitive prices, the market will therefore have the aforementioned closing effect. Especially for potential competitors, the setting of such clauses prevents competitor platforms from reducing the possibility of charging commissions, increasing the cost of potential competitors to enter the market and thus forming a market blocking effect. Of course, it is necessary to consider the scope of goods or services provided by upstream suppliers and the scope of downstream retailers involved in the MFC clause. Only when the market has a large scope will the blockade effect be achieved.

The legislative logic of China’s anti-monopoly law is to protect the basic mechanism of the market economy in order to maintain the competition mechanism and bring benefits to consumers. In fact, whether it is the increase of competition or the improvement of efficiency, it will ultimately improve the well-being of consumers. Similarly, Article 1 of the Anti-Monopoly Law also lists “protecting the interests of consumers” as a legislative purpose.

“The law is an objective right, and a right is a subjective law.” The legal interest evaluation standard focuses on the balance of interests and the balance between operators, competitors and consumers. The value positioning should be at the best balance or combination. Therefore, the anti-monopoly law enforcement agencies should adhere to the protection of legal interests of the anti-monopoly law in the process of law enforcement. When dealing with new issues in the new era and new industries, the conflict between economic efficiency and consumer interests should be properly handled, and handled prudently to reduce the consequences of judicial non-compliance.

## Conclusion

With the rapid development of the Internet, data has shown an explosive growth, and the accumulation of massive data has put forward new requirements for data storage and computing, and various distributed computing and distributed storage systems have emerged one after another. Monopoly is a complex social phenomenon in which economic and legal relations are intertwined, and a structural problem closely related to the core level of social economy. This article mainly searches for H04 patents, and compares the economic efficiency of the TD-SCDMA Patent Alliance. It compares the number of H04 patent applications before and after a company joins the patent alliance, and introduces the relevant content of anti-monopoly and P2P online loan platform. And this paper extracts the fuzzy emotional pattern of users and events, and makes the fuzzy equivalent matrix of lexical emotional tendency and the initial matrix of lexical emotional tendency. Finally, the validity of the fusion algorithm is explained, and the related concepts of vertical integration and the negative effect of restricting competition are introduced. The experimental results show that: after the establishment of the TD-SCDMA Patent Alliance, the number of H04 patents of members has increased rapidly. Although there is a downward trend in the middle, the annual patent application volume is still at a high level. After the establishment of the patent alliance in 2002, the number of H04 patent applications increased by 55%, which shows that the patent alliance can promote the innovation behavior of members in the alliance, and expresses the practical significance of this topic.

## Data availability statement

The original contributions presented in this study are included in the article/supplementary material, further inquiries can be directed to the corresponding author.

## Author contributions

The author confirms being the sole contributor of this work and has approved it for publication.
